# Effect of spontaneous breathing on ventilator-induced lung injury in mechanically ventilated healthy rabbits: a randomized, controlled, experimental study

**DOI:** 10.1186/cc10502

**Published:** 2011-10-21

**Authors:** Jingen Xia, Bing Sun, Hangyong He, Heng Zhang, Chunting Wang, Qingyuan Zhan

**Affiliations:** 1 Department of Respiratory and Critical Care Medicine, Beijing Institute of Respiratory Medicine, Beijing Chao-Yang Hospital, Capital Medical University, 8 Gongren Tiyuchang South Road, Beijing, 100020, PR China

## Abstract

**Introduction:**

Ventilator-induced lung injury (VILI), one of the most serious complications of mechanical ventilation (MV), can impact patients' clinical prognoses. Compared to control ventilation, preserving spontaneous breathing can improve many physiological features in ventilated patients, such as gas distribution, cardiac performance, and ventilation-perfusion matching. However, the effect of spontaneous breathing on VILI is unknown. The goal of this study was to compare the effects of spontaneous breathing and control ventilation on lung injury in mechanically-ventilated healthy rabbits.

**Methods:**

Sixteen healthy New Zealand white rabbits were randomly placed into a spontaneous breathing group (SB Group) and a control ventilation group (CV Group). Both groups were ventilated for eight hours using biphasic positive airway pressure (BIPAP) with similar ventilator parameters: inspiration pressure (P_I_) resulting in a tidal volume (VT) of 10 to 15 ml/kg, inspiratory-to-expiratory ratio of 1:1, positive end-expiration pressure (PEEP) of 2 cmH_2_O, and FiO_2 _of 0.5. Inflammatory markers in blood serum, lung homogenates and bronchoalveolar lavage fluid (BALF), total protein levels in BALF, mRNA expressions of selected cytokines in lung tissue, and lung injury histopathology scores were determined.

**Results:**

Animals remained hemodynamically stable throughout the entire experiment. After eight hours of MV, compared to the CV Group, the SB Group had lower PaCO_2 _values and ratios of dead space to tidal volume, and higher lung compliance. The levels of cytokines in blood serum and BALF in both groups were similar, but spontaneous breathing led to significantly lower cytokine mRNA expressions in lung tissues and lower lung injury histological scores.

**Conclusions:**

Preserving spontaneous breathing can not only improve ventilatory function, but can also attenuate selected markers of VILI in the mechanically-ventilated healthy lung.

## Introduction

Ventilator-induced lung injury (VILI) is one of the most serious complications of mechanical ventilation (MV). The main mechanisms involved are over-distension of alveoli at high lung volume (volutrauma) and cyclic opening and closing of peripheral airways at low lung volume (atelectotrauma) [1]. VILI can result in serious lung parenchymal insults, such as increased permeability of the alveolar-capillary barrier, pulmonary edema, atelectasis, and parenchymal damage [2]. It may also result in the development of inflammatory responses in the local pulmonary and systemic circulations (biotrauma) [3,4], which can then affect the functions of other organs [5,6]. It is commonly accepted that increased production of cytokines, particularly interleukin (IL)-6, IL-1β, IL-10, tumor necrosis factor (TNF)-α, and macrophage inflammatory protein (MIP)-2, plays a key role in initiating or perpetuating lung injury [3-7]. Many clinical studies show that a "lung protective ventilation strategy", which decreases the induction of these cytokines, can remarkably improve patients' clinical outcomes [8-10].

Preserving spontaneous breathing is associated with fewer complications than control ventilation during positive pressure respiratory support, such as by increasing the gas distribution of dependent lung regions [11-14], improving cardiac performance [14-16], promoting ventilation-perfusion matching [14,15,17], preventing diaphragm disuse atrophy [18,19], and decreasing the use of sedative and analgesic drugs [16]. However, the effect of spontaneous breathing on VILI is unknown. Activation of the inspiratory muscles, particularly the diaphragm, can induce greater negative pleural pressures and transalveolar pressures, which can improve the homogenous distribution of ventilation [14], diminish atelectasis [12,20], and thereby reduce lung mechanical stress and strain. However, spontaneous breathing during MV may subsequently induce some conditions that can aggravate lung injury, such as alveolar over-distension caused by increased transalveolar pressure [21], higher pulmonary capillary blood flow caused by increased cardiac output [22], a rapid respiratory rate [23], patient-ventilator asynchrony [24] and others. Recently, several experimental studies showed that preserving spontaneous breathing during mechanical ventilation can attenuate VILI in lung with acute lung injury (ALI) [25-27].

We hypothesized that spontaneous breathing during MV, as compared to control ventilation, would attenuate changes in selected markers of VILI in the healthy lung. To test this hypothesis, we used a rabbit model of the normal lung. Lung injury was evaluated by the levels of inflammatory markers in blood serum and bronchoalveolar lavage fluid (BALF), mRNA expressions of selected cytokines in lung tissues, and lung histopathology examinations.

## Materials and methods

The study was conducted with the approval of the Animal Care Committee of Capital Medical University (Beijing, China), and all animal procedures were carried out in compliance with Institutional Standards for the Care and Use of Laboratory Animals.

### Animals and anesthesia

Our experiments were performed with 24 healthy New Zealand white rabbits, with weights ranging from 2.0 to 2.6 kg. Animals were anesthetized with 3% pentobarbital sodium (Sigma Chemical Co., St. Louis, MO, USA) at 25 mg/kg followed by continuous infusion of 3% pentobarbital sodium at 1 to 2 mg/kg/h. Then, an endotracheal tube (inner diameter of 4 mm) was inserted via tracheotomy. Rabbits were mechanically ventilated (Evita 4, Drager Medical AG & Co., KGaA, Lübeck,Germany) using the biphasic positive airway pressure (BIPAP) mode with baseline ventilator settings: FiO_2 _of 0.5; positive end-expiration pressure (PEEP) of 2 cmH_2_O; inspiration pressure (P_I_) resulting in a tidal volume (VT) of 10 to 15 ml/kg; respiratory rate (RR) of 30 breaths/minute; and inspiratory-to-expiratory (I:E) ratio of 1:1. If necessary, RR was adjusted to maintain PaCO_2 _within 35 to 60 mmHg. If PaCO_2 _was not within this range when RR was 50 breaths/minute, we increased P_I _by 1 to 2 cmH_2_O each time. FiO_2 _and inspiratory-to-expiratory ratio were not changed during the entire experiment. If spontaneous breathing occurred, which was assessed by capnography [28], Pipecuronium Bromide (Gedeon Richter Plc. Budapest, Hungary) at 0.3 mg/kg/h was infused for muscle relaxation.

One 20-gauge catheter was placed in a marginal ear vein for fluid and drug administration. A second catheter was inserted into a carotid artery to monitor blood pressure (BP) and heart rate (HR) and for blood gas sampling. A third catheter was placed in a jugular vein to monitor central venous pressure and for sampling venous blood.

### Experimental protocol

**Figure 1:**
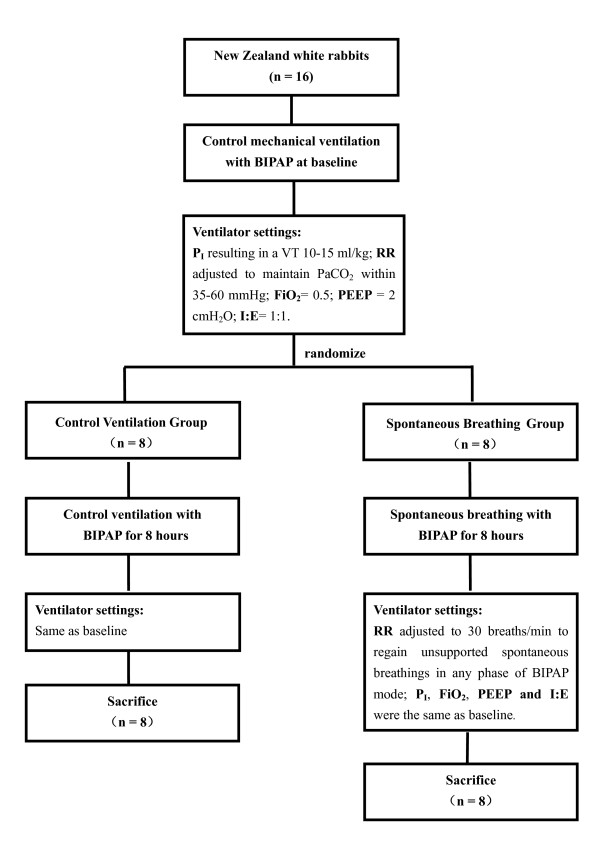
**Flow chart of experiment**. BIPAP, biphasic positive airway pressure; FiO_2_, fraction of inspired oxygen; I:E, inspiratory-to-expiratory ratio; PEEP, positive end-expiration pressure; P_I_, inspiratory pressure; RR, respiratory rate; VT, tidal volume.

After surgical intervention was completed, BP and HR readings that fluctuated by less than 20% were used as baseline. Non-ventilated animals (control animals; *n *= 8) were immediately sampled after sedation and tracheostomized to avoid hypercapnia. The other sixteen rabbits were randomly sorted (opaque sealed envelopes) into a control ventilation group (CV Group; *n *= 8) and a spontaneous breathing group (SB Group; *n *= 8) (Figure 1). Normal saline (0.9%) was administered as a maintenance fluid at the rate of 8 to 10 ml/kg/h. If mean arterial blood pressure was lower than 80 mmHg, fluid boluses of 5 ml/kg of normal saline were administrated. All animals received similar amounts of total IV fluids over eight hours.

After eight hours of MV, all animals were exsanguinated via a carotid artery, and lung tissues and heart were harvested. Bronchoalveolar lavage (BAL) was performed for the left lower lobes. Tissue samples from the left upper lobes were stored in liquid nitrogen for selected cytokine mRNA analyses. Tissue samples of the right four lobes of all animals were immediately fixed in 10% buffered formalin for histological analysis.

### Ventilator setting

**Figure 2:**
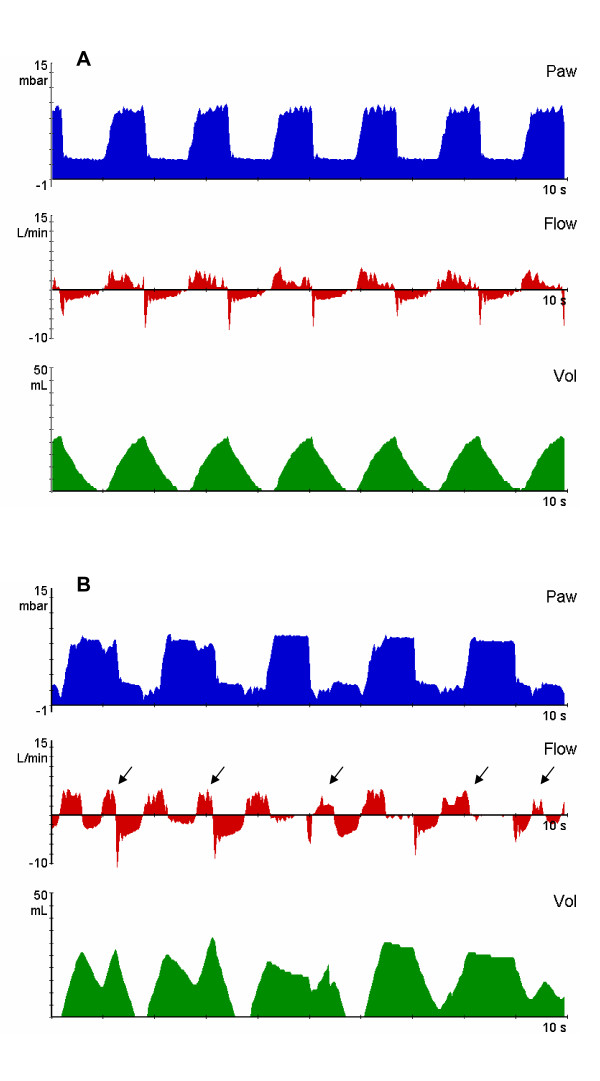
**Representative recordings of airway pressure (Paw), flow and volume (Vol) in experimental groups**. The upper panel (A) shows that the animal has no spontaneous breathing, the BIPAP is equal to PCV. The mechanical RR was 37 breaths/minute, and the Pmean was 5 cmH_2_O. The lower panel (B) shows that non-supported spontaneous breathings (black arrows) were possible during each phase of the ventilatory cycle. The mechanical RR was set at 30 breaths/minute, total RR was 40 breaths/min and the Pmean was also 5 cmH_2_O. A and B had equal I:E ratio (1:1), P_I _(8 cmH_2_O) and PEEP (2 cmH_2_O). BIPAP, biphasic positive airway pressure; I:E, inspiratory-to-expiratory ratio; PCV, pressure control ventilation; PEEP, positive end-expiration pressure; P_I_, inspiratory pressure; Pmean, mean airway pressure; RR, respiratory rate.

For the CV Group, the animals' SB was inhibited continuously and all animals were ventilated with the same baseline ventilator parameters during the entire experiment. For the SB Group, to regain a rabbit's spontaneous breathing, muscle relaxant infusion was stopped and the pentobarbital sodium infusion rate was lowered; then ventilator RR was decreased to 30 breaths/minute to make sure that rabbits could show unsupported spontaneous breathings at high pressure (P_I_) and low pressure (PEEP) levels of BIPAP mode; and the other parameters were not different from baseline ventilator settings, including P_I_, PEEP, I:E ratio and FiO_2 _(Figure 2); the dose of pentobarbital sodium was carefully adjusted to maintain the rate of non-supported spontaneous breathing within 3 to 15 breaths/minute.

Mean airway pressure (Pmean) for the BIPAP mode can be calculated by [29]:

$$\text{mean}\;\text{airway}\;\text{pressure}\;=\;\frac{{\text{P}}_{1}\times {\text{T}}_{1}+\text{PEEP}\times {\text{T}}_{\text{E}}}{{\text{T}}_{1}+{\text{T}}_{\text{E}}}.$$

Here, T_I _is the length of time for which P_I _is maintained; T_E _is the length of time during which PEEP is held. If the ratio of T_I _to T_E _is fixed at 1:1, then the Pmean could be kept constant when we changed the cycle frequency from P_I _to PEEP (or RR). Using this to adjust ventilator parameters (detailed above), we could keep the level of ventilatory support (Pmean) comparable between SB group and CV group of rabbits in our study. Thereafter, the sole difference between the SB group and CV group was whether or not the animals had their spontaneous breathing preserved (Figure 2).

### Measurements and calculations

Arterial pressure, heart rate, central vein pressure and ventilatory parameters were continuously measured each hour. An in-line pressure differential pneumotachometer (CO_2_SMO Puls; Novametrix Medical Systems, Wallingford, CT, USA) was used to measure end-tidal CO_2 _(ETCO_2_), tidal volume, spontaneous tidal volume, spontaneous respiratory rate, total minute volume, spontaneous minute volume, mean airway pressure and peak airway pressure. The ratio of alveolar dead space to tidal volume (VD/VT) was calculated by [30]: VD/VT = (PaCO_2_-ETCO_2_)/PaCO_2_.

At baseline and at the end of eight hours of MV, we measured static lung compliance (Cs). Before measurement, we used Pipecuronium Bromide (0.3 mg/kg) to depress the animals' spontaneous breathing. Thus, the BIPAP mode was equal to PCV mode: Inspiratory pressure (P_I_) and PEEP were not changed; inspiration time was set to five seconds; expiration time was prolonged to allow the expiratory flow to return to zero; and the expiratory tidal volume (VTexp) was recorded. The calculation for Cs used the following formula: Cs = VTexp/(P_I_-PEEP) [31].

Arterial blood gas samples were obtained at baseline, and at one, two, four, six and eight hours after randomization. Arterial blood gas variables were determined by an ABL 725 analyzer (Radiometer, Copenhagen, Denmark), including pH, PaCO_2_, PaO_2_, HCO_3_^- ^, and lactic acid.

### Bronchoalveolar lavage

Sterile normal saline (10 ml) was used to lavage the left lower lobes. After five seconds, the lavage liquid was recycled. The return volume was 5 to 6 ml. These samples were immediately centrifuged at 3,000 to 4,000 rpm for 15 minutes. Supernatant aliquots were kept frozen at -40°C for subsequent analysis.

### Blood measurements

Using ethylenediaminetetraacetic acid (EDTA), venous blood samples (5 ml) from a central vein were obtained at baseline, and at two, four, six and eight hours after randomization. These were immediately centrifuged at 3,000 to 4,000 rpm for 15 minutes. Serum was kept frozen at -40°C for subsequent analysis.

### Cytokine and protein measurements

BALF, serum and lung homogenate concentrations of IL-6, IL-1β, IL-10, TNF-α, and MIP-2 were determined using commercial enzyme-linked immunosorbent assay (ELISA) kits for rabbits (Rapidbio, Calabasas, CA, USA). All ELISAs were done by the same technician according to the manufacturers' guidelines. Total protein levels in BALF were determined using a Bradford Protein Assay Kit (Sun Biomedical Technology, Beijing, China) according to the manufacturers' instructions with BSA as standard.

### Cytokine mRNA analysis by quantitative real-time reverse transcription polymerase chain reaction (RT-PCR)

Total RNA was extracted from lung tissue with a Trizol Isolation Kit (Sun Biomedical Technology) according to the manufacturer's protocol. Initially, the left upper lung tissue maintained in liquid nitrogen was placed in lysis buffer, then immediately disrupted and homogenized using a rotor-stator homogenizer. About 50 to 100 mg of material was used for RNA isolation with 1 ml of Trizol reagent. M-MLV reverse transcriptase (Sun Biomedical Technology) and oligo-(dT) 12 to 18 primers (Sun Biomedical Technology) were used to generate total cDNA. PCR was performed with a reaction volume of 50 μl using a BioEasy SYBR Green I Real Time PCR Kit (Sun Biomedical Technology) according to the manufacturer's instructions. As an internal control, glyceraldehyde-3-phosphate dehydrogenase (GADPH) primers were used for RNA template normalization. The results obtained from real-time RT-PCR were quantified by the 2^-ΔΔCt ^method, as previously reported [32,33]. Each sample was tested in triplicate. The sense (S) and antisense (AS) of the primers (5'-3') used for each cytokine were:

TNF-α: AGCGCCATGAGCACTGAGA/GCCACGAGCAGGAAAGAGAA

IL-6: AGCCCGACTATGAACTCCTTCAC/CCGGATGCTCTCGATGGTT

IL-10: AGAACCACAGTCCAGCCATCA/GCTTGCTGAAGGCGCTCTT

IL-1β: GCAGACGGGAAACAGATTGTG/TTGCCTGAATGGCAGAGGTAA

### Lung histopathology

Samples from the central parts of four different right lobes of the all animals were selected for histopathology examinations. Each sample was sectioned, stained with hematoxylin and eosin, and scored by a pathologist blinded to the experimental design. A lung injury score for each lung section was evaluated using a VILI histopathology scoring system as previously described [34]. The VILI scoring system included four items: alveolar congestion; hemorrhage; infiltration and aggregation of neutrophils in airspace or vessel wall; and thickness of alveolar wall/hyaline membrane formation. The VILI scoring system was graded from 0 (normal lung) to 4 (very severe involvement in > 75% of the lung). Thus, the total score for each section ranged from 0 to 16. The overall lung injury score for each animal was the average score of the four sections from each right lobe.

### Statistical analysis

Data are given as mean ± SD or median (interquartile range), as appropriate. The normality of data distributions was assessed with a Kolmogorov-Smirnov test, and the homogeneity of variances was tested with Levene's test. Differences among groups were analyzed by one-way analysis of variance (ANOVA). Continuous variables, including hemodynamics, blood gases, respiratory parameters and cytokines level, were compared using repeated measures ANOVA. Logarithmic transformations of cytokine levels in BALF and serum were made before using parametric tests. *P-*values < 0.05 were considered significant. All analyses used SPSS 11.5 (SPSS Inc., Chicago, IL, USA).

## Results

### Hemodynamics and gas exchange

The mean arterial blood pressures (MBP) within both the control ventilation (CV) and spontaneous breathing (SB) groups were similar and were maintained at the expected levels during the entire experiment (Table 1). Heart rate and central venous pressure remained stable during the experiment, and there were no differences for these two variables between SB group and CV group (Table 1). Both groups received equal administrations of fluid boluses. No other drugs were needed to maintain hemodynamic conditions.

**Table 1:** Physiology response to spontaneous breathing (SB Group) and control ventilation (CV group).

	Time = 0 h			Time = 8 h		
	CV group (*n *= 8)	SB group (*n *= 8)	*P_1_*	CV group (*n *= 8)	SB group (*n *= 8)	*P_2_*
HR (breaths/minute)	232.2 ± 23.7	239.3 ± 28.2	0.66	219.3 ± 19.8	235.2 ± 21.7	0.22
MBP (mmHg)	89.0 ± 11.0	91.4 ± 16.5	0.75	92.6 ± 17.8	90.7 ± 9.4	0.81
CVP (mmHg)	3.6 ± 0.7	3.7 ± 0.7	0.74	3.6 ± 0.5	3.9 ± 0.6	0.41
VT (ml/kg)	12.9 ± 0.8	12.3 ± 1.7	0.54	12.2 ± 1.4	13 ± 0.6	0.24
VTspont (ml/kg)	0	0	-	0	10.9 ± 0.8	-
RR_TOT _(breaths/minute)	38.3 ± 4.1	37.5 ± 2.7	0.69	45.0 ± 4.5	39.5 ± 7.4	0.15
RRspont (breaths/minute)	0	0	-	0	8.8 ± 5.4	-
MV_TOT _(L/minute)	1.26 ± 0.28	1.04 ± 0.23	0.18	1.50 ± 0.26	1.18 ± 0.33	0.10
P_I _(cmH_2_O)	8 ± 1.6	8 ± 1.5	1.00	9 ± 0.6	8.3 ± 1.8	0.43
Pmean (cmH_2_O)	4.67 ± 0.8	4.67 ± 0.5	1.00	5.33 ± 0.8	5.00 ± 0.6	0.45
Compliance(ml/cmH_2_O)	5.4 ± 0.7	5.55 ± 2.6	0.87	4.3 ± 0.8	7.6 ± 2.4*	0.01
Arterial pH	7.34 ± 0.08	7.38 ± 0.06	0.27	7.24 ± 0.05*	7.33 ± 0.08	0.04
PaCO_2 _(mmHg)	48.5 ± 8.7	43.9 ± 6.2	0.32	51.5 ± 7.8	38.2 ± 7.1*	0.01
PaO_2_/FiO_2 _(mmHg)	399.0 ± 46.4	430.3 ± 40.0	0.24	437.7 ± 60.3	476.3 ± 11.6	0.40
Lactic acid (mmol/L)	1.0 ± 0.2	1.4 ± 0.4	0.10	2.1 ± 0.4	1.7 ± 0.7	0.70

Results are given as mean ± SD. *P < 0.05 compared with baseline within the same group. *P_1_*, compared between SB group and CV group at 0 h; *P_2_*, compared between SB group and CV group at 8 h. CVP, central venous pressure; FiO_2_, fraction of inspired oxygen; HR, heart rate; MBP, mean arterial pressure; MV_TOT_, total minute volume; PaCO_2_, arterial partial pressure of carbon dioxide; PaO_2_, arterial partial pressure of oxygen; P_I_, inspiratory pressure; Pmean, mean airway pressure; RRspont, non-supported spontaneous respiratory rate; RR_TOT_, total respiratory rate; VT, tidal volume; VTspont, tidal volume of non-supported spontaneous breathing.

**Figure 3:**
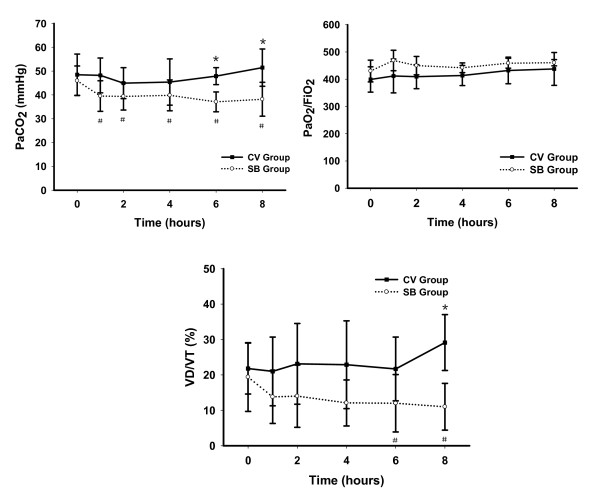
**Time course for PaCO_2 _, PaO_2_/FiO_2 _and VD/VT in experimental groups**. Results are given as mean ± SD. *P < 0.05 between groups at the same time. **^#^***P *< 0.05 compared with baseline within the same group. CV, control ventilation; FiO_2_, fraction of inspired oxygen; PaCO_2_, arterial partial pressure of carbon dioxide; PaO_2_, arterial partial pressure of oxygen; SB, spontaneous breathing; VD/VT, ratio of alveolar dead space to tidal volume.

The pH values of all animals were greater than 7.2 during the experiment without the administration of sodium bicarbonate; however, after eight hours of ventilation, the SB group had a higher mean pH value (7.33 ± 0.08) than the CV group (7.24 ± 0.05) (Table 1). PaCO_2 _values of all animals were maintained within the range noted in Methods, although PaCO_2 _values were higher in the CV group compared to the SB group after randomization, and this difference was significantly different between the groups after six hours of ventilation; PaCO_2 _values in the SB group reduced significantly after randomization (*P *< 0.05; Figure 3). Both groups had similar PaO_2_/FiO_2 _ratios (Figure 3).

The total respiratory frequency (RR_TOT_) and total minutes of ventilation (MV_TOT_) were stably maintained for both groups of rabbits, with no significant differences between the groups (Table 1). However, the ratio of dead space to tidal volume in the SB group decreased gradually after randomization, and showed a significant difference between SB group and CV group after eight hours (Figure 3).

### Respiratory mechanics

Rabbits in the SB group and the CV group had similar Pmean and P_I _throughout the experiment (Table 1). Both groups had similar static lung compliances (Cs) at baseline. However, the Cs values of the SB group markedly increased and were significantly higher than in the CV group after eight hours of ventilation (Table 1). Conversely, the Cs values of the CV group slightly decreased by the end of the experiment.

### Total protein levels in BALF

**Figure 4:**
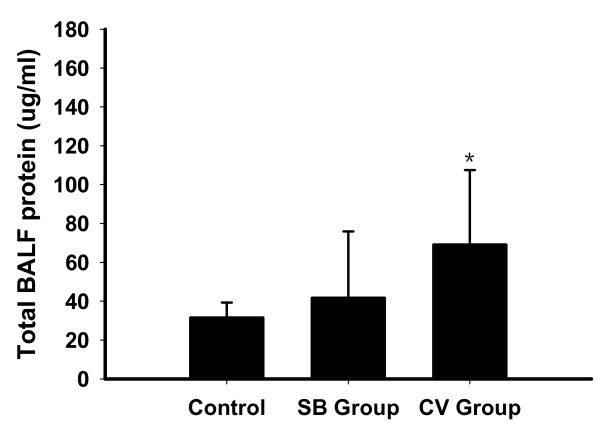
**Total BALF protein in non-ventilated (control) and experimental groups**. Results are given as mean ± SD.*P < 0.05 compared with control animals. BALF, bronchoalveolar lavage fluid; CV, control ventilation; SB, spontaneous breathing.

Total BALF protein levels in CV group were significantly higher as compared with control animals (*P *< 0.05; Figure 4). No significant difference was found between the SB group and control animals. Total BALF protein levels were slightly lower in the SB group than in the CV group (41.8 ± 34.1 vs 69.2 ± 38.3 μg/ml, *P *= 0.112).

### Cytokine levels in serum, bronchoalveolar lavage fluid (BALF) and lung homogenate

**Figure 5:**
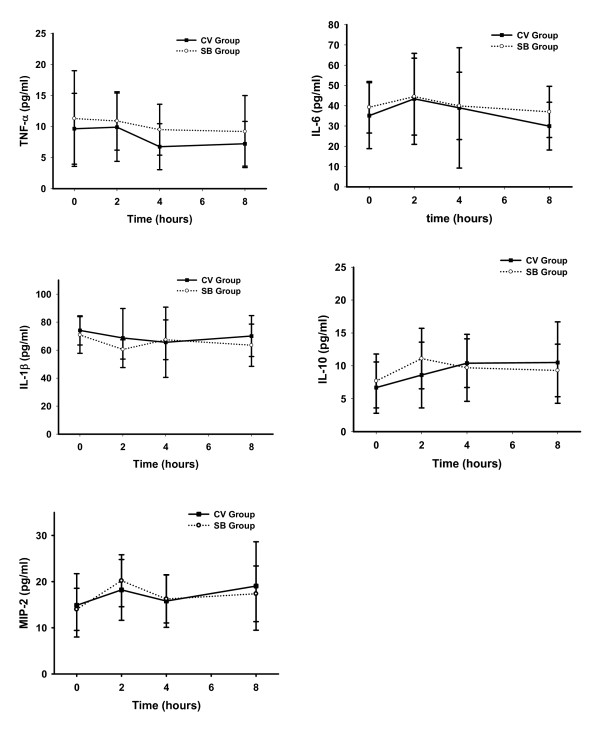
**Time course for the serum concentration of different cytokines in experimental groups**. Results are given as mean ± SD. CV, control ventilation; SB, spontaneous breathing.

**Figure 6:**
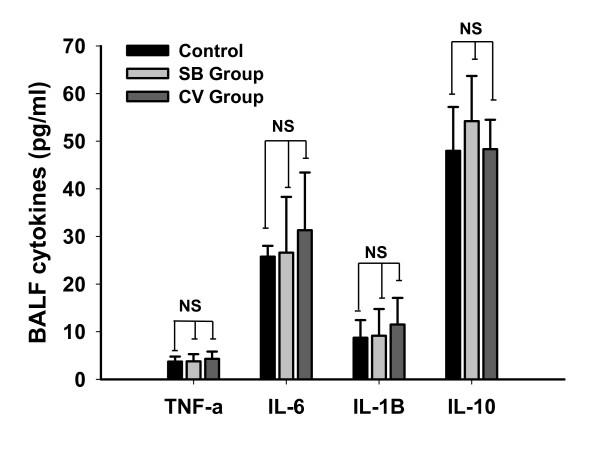
**Concentrations of different cytokines in bronchoalveolar lavage fluid in nonventilated (control) and experimental groups**. Results are given as mean ± SD. BALF, bronchoalveolar lavage fluid; CV, control ventilation; NS, non-significant difference among three groups; SB, spontaneous breathing.

**Figure 7:**
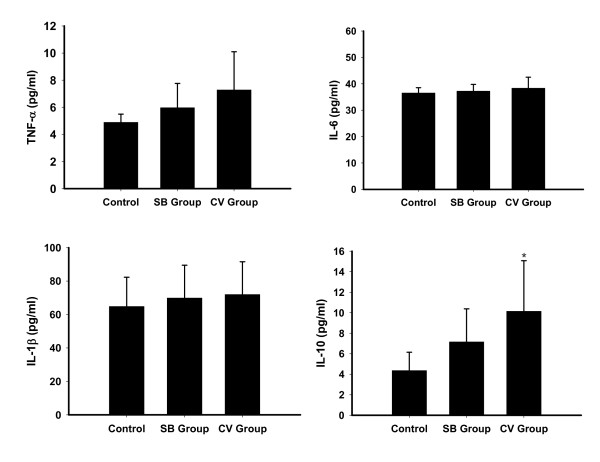
**Pulmonary level of different cytokines in nonventilated (control) and experimental groups**. Results are given as mean ± SD. *P < 0.05 compared with control animals. CV, control ventilation; SB, spontaneous breathing;

There were no differences in serum TNF-α, IL-6, IL-1β, IL-10 or MIP-2 concentrations between the SB group and CV group during the entire experiment (Figure 5). There were also no significant differences within each group at baseline and at the end of the experiment. We also did not find any differences between the CV group and SB group for any of the cytokines in BALF (TNF-α, 4.29 ± 1.52 vs 3.78 ± 1.5 pg/ml; IL-6, 31.3 ± 12.1 vs 26.6 ± 11.7 pg/ml; IL-1β, 11.5 ± 5.6 vs 9.16 ± 5.6 pg/ml; IL-10, 48.34 ± 6.17 vs 54.2 ± 9.5 pg/ml) (Figure 6). The cytokines (TNF-α, IL-6 and IL-1β) level of lung homogenates in the CV group and the SB group were slightly higher than control animals (*P *> 0.05), but IL-10 concentrations in the CV group were significantly higher than control animals (*P *< 0.05). Pulmonary levels of IL-6, IL-1β, IL-10 and TNF-α between the CV group and the SB group were comparable (Figure 7).

### Cytokines' mRNA expressions in lung tissue

**Figure 8:**
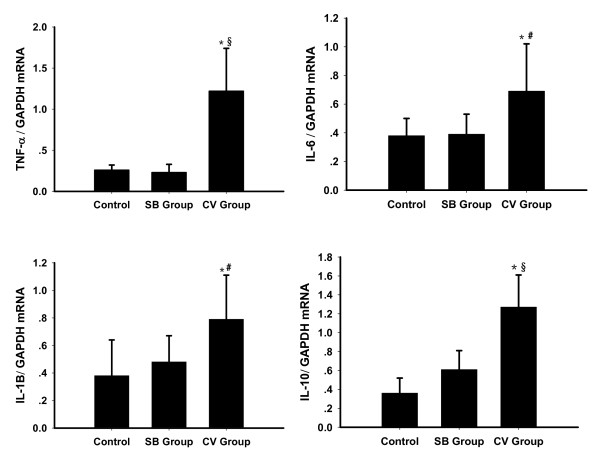
**Cytokine mRNA expression level in nonventilated (control) and experimental groups**. Results are given as mean ± SD. Raw data were normalized to GAPDH expression. *P < 0.05 compared with control group. *P < 0.01 compared between CV group and SB group. **^# ^***P *< 0.01 compared between CV group and SB group. CV, control ventilation; GADPH, glyceraldehyde-3-phosphate dehydrogenase; SB, spontaneous breathing.

The expressions of IL-6, IL-1β, IL-10 and TNF-α mRNA in lung tissues were evidently higher in the CV group compared to the SB group and control animals (*P *< 0.05; Figure 8). The TNF-α mRNA expression was six times higher in the CV group than in the SB group (*P *= 0.001). The other cytokines' mRNA expressions were about two times higher in the CV group as compared to the SB group. No significant difference was found between the SB group and control animals.

### Lung histopathological injury

**Figure 9:**
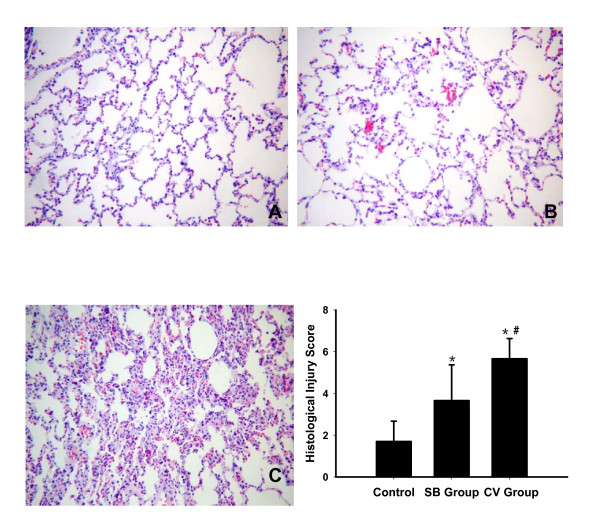
**Representative photomicrographs of the right lower lobe and the lung injury score from nonventilated (control) and experimental groups**. The upper panel shows photomicrographs of hematoxylin-eosin-stained lung sections (magnification ×100) of right lower lobe in control animals (**A**), SB group (**B**) and CV group (**C**). SB group (B) with mild alveolar congestion. CV group (C) with increased alveolar collapse and congestion, thickening of alveolar septum, alveolar capillary congestion, and inflammatory cell migration. The lower right panel shows the gradation of the VILI scoring system (detailed in Materials and methods) in SB group and CV group. Results are given as mean ± SD. *P < 0.05 compared with control groups. **^# ^***P *< 0.01 compared between CV group and SB group. CV, control ventilation; SB, spontaneous breathing; VILI, ventilator-induced lung injury.

Gross histological evaluations of the right lower lobe lungs showed that the SB group had nearly normal lung tissue, whereas the CV group showed greater alveolar collapse, thickness of the alveolar septum, alveolar capillary congestion, and inflammatory cell migration (Figure 9). Also, the average lung histological injury score of the whole right lung was higher in the CV group (5.7 ± 1.0) as compared to the SB group (3.7 ± 1.7, *P *= 0.005) and control animals (1.7 ± 1.0, *P *< 0.001; Figure 9). The average lung histological injury score were also higher in the SB group than in control animals (*P *= 0.005).

## Discussion

To our knowledge, this is the first study on the effects of spontaneous breathing on VILI in the mechanically-ventilated healthy lung. We found that preserving spontaneous breathing during MV could not only improve ventilatory function, but could also attenuate selected markers of VILI.

For our experiments, we selected tidal volume of as large as 10 to 15 ml/kg to ventilate animals with healthy lungs. Hong *et al*. [33] found that a moderate tidal volume (15 ml/kg) and low PEEP (3 cmH_2_O) resulted in lower inflammatory response expressions in lung tissue and lung injury than with a small tidal volume (6 ml/kg) and high PEEP (10 cmH_2_O) in a healthy animal model. Thus, we set PEEP to 2 cmH_2_O to avoid excessive distension of alveoli and concomitantly prevent alveolar collapse at end-expiration. In addition, we applied a biphasic positive airway pressure (BIPAP) mode during MV, which allowed animals to breathe freely at any phase of the mechanical ventilatory cycle. When rabbits did not exhibit spontaneous breathing, the BIPAP mode was equal to the pressure control ventilation (PCV) mode. Pmean, which reflects the average level of alveolar pressure during the entire respiratory cycle, is also an important factor contributing to the development of lung injury [35]. In our study, the Pmean between the SB group and the CV group was comparable by the method of adjusting ventilator settings (detailed in Materials and methods).

We demonstrated that control ventilation caused higher dead space ventilation (VD/VT) than preserving spontaneous breathing during positive pressure MV. Putensen *et al*. [15] also found that spontaneous breathing could improve dead space ventilation in mechanically-ventilated patients with acute respiratory distress syndrome. This phenomenon may be explained by considering that preserving respiratory muscle contraction favors more ventilation to dependent lung regions [12-14], reduces atelectasis [12,20], and improves ventilation-perfusion matching [14,15]. On histopathology examinations, we also found that more alveolar collapse appeared in our CV group.

Furthermore, we showed that preserving spontaneous breathing was followed by a lower PaCO_2 _than control ventilation after eight hours of MV, while other studies [15,17] did not find differences between the SB and CV groups in PaCO_2_. These differences may be attributed to the number of hours of control ventilation. The previous studies used only two hours of control ventilation, while our study used eight hours. We also did not find any significant differences in the first two hours of MV after randomization, however, after six hours, there was a difference and this was notable.

Although spontaneous breathing could obviously improve ventilatory function after a long time of MV, there was no difference between the SB and CV groups on oxygenation, which is in contrast to other reports [12,15]. Wrigge *et al*. [12] found that preserving spontaneous breathing resulted in better oxygenation compared to control ventilation in an acute lung injury model with MV. It is possible that there is less alveolar collapse and lung injury in a normal lung model than an acute lung injury model.

Preserving spontaneous breathing can reduce atelectasis and improve gas distribution in the ventilatory-supported normal lung. However, prior to this study, we could not clearly identify the effects of spontaneous breathing on VILI in the normal lung. In our study, we demonstrated that there were no differences between the SB group and the CV group in the concentrations of all the measured cytokines in serum and BALF. However, animals with spontaneous breathing showed significantly lower gene expression levels of inflammatory cytokines (TNF-α, IL-6, IL-1β) and anti-inflammatory cytokine (IL-10) in lung tissues. Furthermore, the gene expression levels of cytokines in SB group were similar to control animals. The level of IL-10 in the SB group was parallel decreased, since it has anti-inflammatory properties. It was suggested that the inflammatory response was significantly reduced in lung tissues. TNF-α mRNA expression was six times higher in our CV group than in our SB group. The mRNA levels of other cytokines (IL-6, IL-1β, and IL-10) were about two times higher in the CV group compared to the SB group. Moreover, we also found total protein levels in BALF were slightly lower in the SB group than in the CV group. Maybe cytokines were already expressed in alveolar epithelial cells, but had not yet migrated into the alveolar lumen or systemic circulation, because we found that the cytokines level in lung homogenate increased slightly in the CV group, especially anti-inflammatory cytokine (IL-10). With an extended time of control MV, inflammatory responses may be further aggravated and lead to increased cytokine levels in the alveolar lumen and systemic circulation.

Consistent with the inflammatory responses, the histopathological damage to lung tissue was more severe in animals with control ventilation than in those with spontaneous breathing. To compare lung injury of the whole right lung of all animals, we selected the central parts of four different right lobes for histopathology examination and did not divide the right lung into non-dependent and dependent parts. The pathological changes in the animals of our CV group agree with the changes seen in healthy animals with similar ventilator settings in other studies [33,36]. Control ventilation can cause more alveolar collapse and congestion, thickening of the alveolar septum, and infiltrations of inflammatory cells into lung tissue. Lung histological scores also showed that lung injury was more severe in animals with control ventilation. Our histological findings were in accord with previous studies [26,27] which demonstrated that the assisted ventilation mode could reduce lung injury compared to the control ventilation mode in an acute lung injury model.

There are many factors that can attenuate VILI when spontaneous breathing is preserved during MV with a healthy lung: increased ventilation to dependent lung regions caused by an increase of transalveolar pressure, reduced occurrence of atelectasis, increased recruited lung volume [17,20], improved compliance and further avoidance of atelectotrauma caused by the opening and closing of distal airways during tidal breaths [37,38]; more homogeneous distribution of gas, less dead space ventilation and hyperinflation [12,17]; redistribution of blood in alveolar capillaries, improved ventilation and perfusion matching [14]; variability of breathing patterns [25]; and physiological feedback and intrinsic defense mechanisms during spontaneous breathing, such as the Hering-Breuer reflex, may further prevent VILI.

Our study had several limitations. First, because our study animal model was healthy rabbits, we cannot directly extend our results to normal human lung function. Second, a larger VT of 10 to 15 ml/kg was selected in our study. It can cause lung injury in a previously healthy lung, as showed in CV group animals of our study. Therefore, it is necessary to design another similar study using smaller VT of 6 to 7 ml/kg to testify the protective effect of spontaneous breathing on VILI in healthy animals. Third, PaCO_2 _values were different between the SB group and the CV group, which could affect the experimental results of lung injury. Clinical and animal studies have shown that hypercapnic conditions had a protective effect against lung injuries, which were associated with significantly improved pulmonary edema, increased pulmonary compliance, and reduced levels of cytokines [39-41]. However, the CV group, with a higher PaCO2 than the SB group, did not exhibit a lower inflammatory response. It was further suggested that spontaneous breathing had a protective effect against VILI. Fourth, some invasive procedures can also have an impact on cytokine levels; thus, to guarantee comparable cytokine levels at baseline, surgical interventions were administrated by fixed researchers, who strictly controlled the time for our procedures and the amount of blood loss. Fifth, we did not measure transalveolar pressure to evaluate respiratory effort. Finally, SB group animals used less muscle relaxant (Pipecuronium Bromide) and anesthesia (Pentobarbital sodium). Unfortunately, it remains unknown if they can directly affect inflammatory responses.

## Conclusions

In the present experimental study, we show that preserving spontaneous breathing during MV can not only improve ventilatory function, but can also attenuate selected markers of VILI in the healthy lung. However, our results cannot be extrapolated to humans, and clinical trials are necessary to confirm our results in humans.

## Key messages

• Assuming comparable levels of ventilation support, preserving spontaneous breathing during MV is associated with better respiratory function as compared to control ventilation: both a lowered ratio of dead space ventilation to tidal volume and PaCO_2_.

• Compared to control ventilation, preserving spontaneous breathing can result in better lung compliance after eight hours of MV.

• Spontaneous breathing may have an important protective effect against VILI in mechanically ventilated healthy lung.

## Abbreviations

ALI: acute lung injury; BAL: bronchoalveolar lavage; BALF: bronchoalveolar lavage fluid; BIPAP: biphasic positive airway pressure; BP: blood pressure; CV: control ventilation; CPAP: continuous positive airway pressure; Cs: static lung compliance; EDTA: ethylenediaminetetraacetic acid; ETCO2: end-tidal CO2; FiO2: fraction of inspired oxygen; GADPH: glyceraldehyde-3-phosphate dehydrogenase; HR: heart rate; IL: interleukin; MIP-2: macrophage inflammatory protein; mRNA: messenger ribonucleic acid; MV: mechanical ventilation; MV_TOT_: total minute ventilation; PaCO_2_: arterial partial pressure of carbon dioxide; PaO_2_: arterial partial pressure of oxygen; PCV: pressure control ventilation; PEEP: positive end-expiration pressure; P_I_: inspiration pressure; P_high_: high level continuous positive airway pressure; P_low_: low level continuous positive airway pressure; Pmean: mean airway pressure; RT-PCR: real time reverse transcription polymerase chain reaction; RR: respiratory rate; RR_TOT_: total respiratory rate; SB: spontaneous breathing; TNF-a: tumor necrosis factor alpha; VILI: ventilator-induced lung injury; (VD/VT)alv: the ratio alveolar dead space to tidal volume; VT: tidal volume.

## Competing interests

The authors declare that they have no competing interests.

## Authors' contributions

JX participated in the design of the study and in the experiments, was involved in the data extraction as well as statistics, and drafted the manuscript. BS participated in the design of the study and helped to draft the manuscript. HH participated in the design of the study and in the experiments, and helped to draft the manuscript. HZ and CW participated in the design of the study and in the experiments. QZ participated in the design of the study, was involved in interpretation of the results and helped to draft the manuscript. All authors read and approved the final manuscript for publication.
